# Supplementary material for: A quantitative test of the “Ecomorphotype Hypothesis” for fossil true seals (Family Phocidae)
Source: PeerJ. 2024 Jun 19;12:e17592. doi: 10.7717/peerj.17592 (PMC11193399; doi:10.7717/peerj.17592)
Supplement: Supplemental Information 1 [file peerj-12-17592-s001.docx]

**Supplemental Information for: A quantitative test of the “Ecomorphotype Hypothesis” for fossil true seals (Family Phocidae)**

**
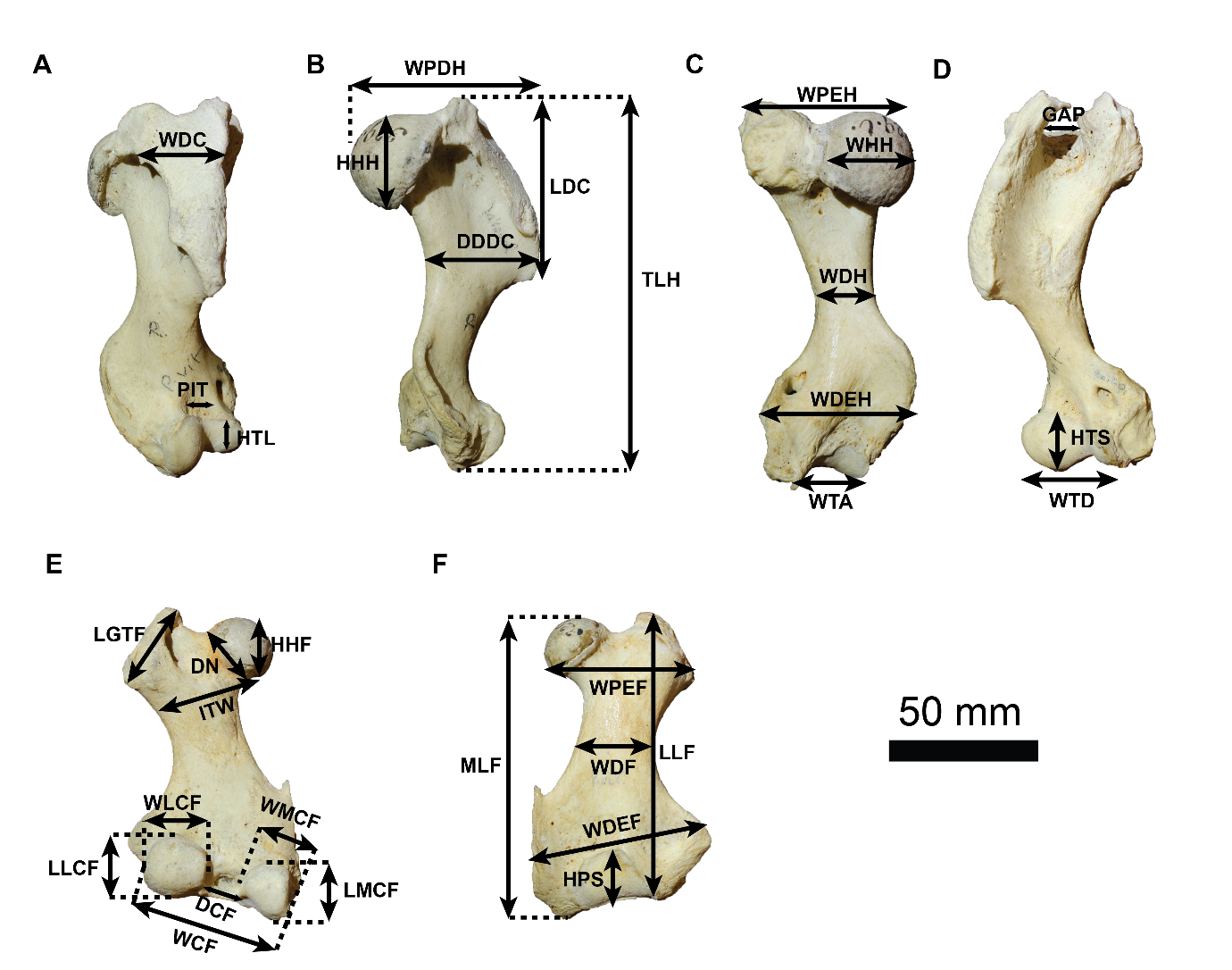
**

**Supplementary Figure 1.** Measurement protocol from Churchill and Uhen (2019). Right humerus (A, B, C, D) and left femur (E, F) of *Phoca vitulina* (NHMUK 329i) in oblique lateral (A), lateral (B), posterior (C, E), and anterior (D, F) views. Definitions of Humerus measurements: DDDC – diaphysis diameter at deltopectoral crest; HHH – proximo-distal height of humeral head; HTL – proximo-distal height of capitulum; HTS – proximo-distal height of trochlea; LDC – proximo-distal deltopectoral crest length; TLH – total proximo-distal length of humerus; WDC – medio-lateral width of deltopectoral crest; WDEH – medio-lateral width of distal epiphysis; WDH – minimum medio-lateral width of diaphysis; WHH – maximum width of humeral head; WPDH - width of proximal epiphysis head to deltopectoral crest; WPEH – width of humeral head to lesser tubercle; WTA – medio-lateral width of trochlea in anterior view; WTD – medio-lateral width of trochlea in posterior view; GAP – width of inter-tubercle space; PIT – anterior-posterior depth of coronoid fossa. Definitions of Femur measurements: DCF – medio-lateral distance between condyles; DN – proximo-distal diameter of the femur neck; HHF – proximo-distal height of femoral head; HPS – proximo-distal height of patellar surface; ITW – medio-lateral intertrochlear width of femur; LGTF – maximum length of the greater trochanter; LLF – maximum proximo-distal length of the lateral femur; MLF – maximum proximo-distal length of the medial femur; LMCF – proximo-distal length of the medial condyle; LLCF – proximo-distal length of the lateral condyle; WCF – medio-lateral width across condyles; WDEF – maximum medio-lateral width across distal diaphysis; WDF – minimum medio-lateral width of diaphysis; WPEF – maximum medio-lateral width across proximal diaphysis; WLCF – medio-lateral width of lateral condyle; WMCF – medio-lateral width of medial condyle.

**Supplemental Table 1.** List of new specimens measured for this study and Churchill & Uhen 2019. Except where indicated, both humeri and femora were measured for each specimen, and both left and right sides (except for Churchill & Uhen 2019). NHMUK: Natural History Museum, London. NMNS: National Museum of Nature and Science, Tokyo. IRSNB: Royal Belgian Institute of Natural Sciences, Brussels.

| **Species** | **Specimen number** | **Notes** | **Ecomorphotype** |
| --- | --- | --- | --- |
| *Phoca vitulina* | NHMUK 1868.3.21.1 | Sex unknown. | 2 |
| *Phoca vitulina* | NHMUK 329.i | Left humerus missing. Sex unknown. | 2 |
| *Pusa hispida* | NHMUK ZD 1997.585 | Right femur missing. Sex unknown. | 2 |
| *Pusa hispida* | NHMUK 1938.12.10.5 | Female. | 2 |
| *Pusa sibirica* | NHMUK 1965.9.6.2 | Male. | 2 |
| *Pusa sibirica* | NHMUK 1960.12.21.1 | Female. | 2 |
| *Pusa sibirica* | NHMUK 1965.9.6.1 | Female. | 2 |
| *Histriophoca fasciata* | NHMUK 1966.12.7.2 | Male. | 3 |
| *Pagophilus groenlandicus* | NHMUK 1846.6.18.3 | Sex unknown. | 2 |
| *Pagophilus groenlandicus* | NHMUK 1938.12.10.1 | Female. | 2 |
| *Pagophilus groenlandicus* | NHMUK 1951.11.28.2 | Male. | 2 |
| *Halichoerus grypus* | NHMUK ZD 2003.361 | Right side only. Sex unknown. | 4 |
| *Halichoerus grypus* | NHMUK 1938.3.12.1 | Left side only. Female. | 4 |
| *Halichoerus grypus* | NHMUK 1956.9.26.1 | Left side only. Female. | 4 |
| *Halichoerus grypus* | NHMUK 1956.9.26.4 | Right side only. Male. | 4 |
| *Halichoerus grypus* | NHMUK 1962.3.6.1 | Left side only. Male. | 4 |
| *Halichoerus grypus* | NHMUK 1951.11.28.1 | Female. | 4 |
| *Halichoerus grypus* | NHMUK 1956.9.26.2 | Right side only. Male. | 4 |
| *Erignathus barbatus* | NHMUK 1887.9.28.1 | Sex unknown. | 1 |
| *Phoca largha* | NMNS M62796 | Right side only. Sex unknown. | 4 |
| *Phocanella pumila* | Cast: NHMUK PV M1199, original: IRSNB 1080-M227 | Right humerus only. Fossil specimen. | Treated as unknown |
| *Phocanella minor* | Cast: NHMUK PV M1206, original: IRSNB 1101-M234 | Right humerus only. Fossil specimen. | Treated as unknown |
| *Nanophoca vitulinoides* | Cast: NHMUK PV M1212, original: IRSNB 1063-M242 | Left humerus only. Fossil specimen. | Treated as unknown |
| *Nanophoca vitulinoides* | Cast: NHMUK PV M1216, original: IRSNB 1049-M247 | Left femur only. Fossil specimen. | Treated as unknown |
| *Erignathus barbatus* | USNM 16116 | Side and sex unknown. Churchill & Uhen 2019. | 1 |
| *Erignathus barbatus* | USNM 269126 | Side and sex unknown. Churchill & Uhen 2019. | 1 |
| *Erignathus barbatus* | USNM 500250 | Side and sex unknown. Churchill & Uhen 2019. | 1 |
| *Erignathus barbatus* | AMNH 19347 | Side unknown. Male. Churchill & Uhen 2019. | 1 |
| *Erignathus barbatus* | AMNH 28 | Side and sex unknown. Churchill & Uhen 2019. | 1 |
| *Histriophoca fasciata* | USNM 504959 | Side and sex unknown. Churchill & Uhen 2019. | 3 |
| *Histriophoca fasciata* | USNM 504960 | Side and sex unknown. Churchill & Uhen 2019. | 3 |
| *Histriophoca fasciata* | USNM 571367 | Side and sex unknown. Churchill & Uhen 2019. | 3 |
| *Histriophoca fasciata* | AMNH 130246 | Side and sex unknown. Churchill & Uhen 2019. | 3 |
| *Histriophoca fasciata* | AMNH130245 | Side unknown. Male. Churchill & Uhen 2019. | 3 |
| *Pagophilus groenlandica* | USNM 3517 | Side and sex unknown. Churchill & Uhen 2019. | 2 |
| *Pagophilus groenlandica* | USNM 21535 | Side and sex unknown. Churchill & Uhen 2019. | 2 |
| *Pagophilus groenlandica* | USNM 188766 | Side unknown. Male. Churchill & Uhen 2019. | 2 |
| *Pagophilus groenlandica* | AMNH 180016 | Side and sex unknown. Churchill & Uhen 2019. | 2 |
| *Phoca vitulina* | USNM 49911 | Side and sex unknown. Churchill & Uhen 2019. | 2 |
| *Phoca vitulina* | USNM 21056 | Side and sex unknown. Churchill & Uhen 2019. | 2 |
| *Phoca vitulina* | USNM 15276 | Side and sex unknown. Churchill & Uhen 2019. | 2 |
| *Phoca vitulina* | USNM 504298 | Side and sex unknown. Churchill & Uhen 2019. | 2 |
| *Phoca vitulina* | USNM 504299 | Side and sex unknown. Churchill & Uhen 2019. | 2 |
| *Phoca vitulina* | AMNH 15900 | Side and sex unknown. Churchill & Uhen 2019. | 2 |
| *Phoca vitulina* | AMNH 232416 | Side unknown. Male. Churchill & Uhen 2019. | 2 |
| *Phoca vitulina* | AMNH 232402 | Side and sex unknown. Churchill & Uhen 2019. | 2 |
| *Phoca vitulina* | USNM 250712 | Side and sex unknown. Churchill & Uhen 2019. | 2 |
| *Phoca vitulina* | USNM 283568 | Side and sex unknown. Churchill & Uhen 2019. | 2 |
| *Halichoerus grypus* | USNM 218323 | Side and sex unknown. Churchill & Uhen 2019. | 4 |
| *Halichoerus grypus* | USNM 446405 | Side and sex unknown. Churchill & Uhen 2019. | 4 |
| *Halichoerus grypus* | USNM 446406 | Side and sex unknown. Churchill & Uhen 2019. | 4 |
| *Pusa hispida* | USNM 16106 | Side and sex unknown. Churchill & Uhen 2019. | 2 |
| *Pusa hispida* | USNM 7102 | Side and sex unknown. Churchill & Uhen 2019. | 2 |
| *Pusa hispida* | USNM 504208 | Side and sex unknown. Churchill & Uhen 2019. | 2 |
| *Pusa sibirica* | AMNH 185595 | Side and sex unknown. Churchill & Uhen 2019. | 2 |
| *Pusa caspica* | USNM 341615 | Side unknown. Male. Churchill & Uhen 2019. | 2 |
| *Phocanella pumila* | USNM 329059 | Humerus only, side unknown. Churchill & Uhen 2019. | Treated as unknown |
| *Phocanella pumila* | USNM 171151 | Humerus only, side unknown. Churchill & Uhen 2019. | Treated as unknown |
| *Phocanella pumila* | USNM 305283 | Femur only, side unknown. Churchill & Uhen 2019. | Treated as unknown |
| *Phocanella pumila* | USNM 329060 | Femur only, side unknown. Churchill & Uhen 2019. | Treated as unknown |
| *Phocanella pumila* | USNM 175217 | Femur only, side unknown. Churchill & Uhen 2019. | Treated as unknown |
| *Phocanella pumila* | USNM 181649 | Femur only, side unknown. Churchill & Uhen 2019. | Treated as unknown |
| *Phocanella pumila* | USNM 481569 | Femur only, side unknown. Churchill & Uhen 2019. | Treated as unknown |
| *Cryptophoca maeotica* | USNM 214979, cast of LPB 259 | Femur only, side unknown. Churchill & Uhen 2019. | Treated as unknown |
| *Leptophoca "amphiatlantica"* | USNM 321926 | Femur only, side unknown. Churchill & Uhen 2019. | Treated as unknown |
| *Leptophoca proxima* | USNM 559330 | Femur only, side unknown. Churchill & Uhen 2019. | Treated as unknown |
| *Monachopsis pontica* | USNM 214967, cast of LPB 21 | Femur only, side unknown. Churchill & Uhen 2019. | Treated as unknown |
| *Praepusa vindobonensis* | USNM 214993, cast of LPB 158? | Femur only, side unknown. Churchill & Uhen 2019. | Treated as unknown |
| *Praepusa*? *pannonica* | USNM 214978, cast of LPB 5? | Femur only, side unknown. Churchill & Uhen 2019. | Treated as unknown |
